# MIR retrotransposons link the epigenome and the transcriptome of coding genes in acute myeloid leukemia

**DOI:** 10.1038/s41467-022-34211-x

**Published:** 2022-10-31

**Authors:** Aristeidis G. Telonis, Qin Yang, Hsuan-Ting Huang, Maria E. Figueroa

**Affiliations:** 1 Department of Biochemistry and Molecular Biology, University of Miami Miller School of Medicine, Miami, FL USA; 2 Sylvester Comprehensive Cancer Center, University of Miami Miller School of Medicine, Miami, FL USA

**Keywords:** Acute myeloid leukaemia, Cancer epigenetics, Cancer genomics, Acute myeloid leukaemia

## Abstract

*DNMT3A* and *IDH1/2* mutations combinatorically regulate the transcriptome and the epigenome in acute myeloid leukemia; yet the mechanisms of this interplay are unknown. Using a systems approach within topologically associating domains, we find that genes with significant expression-methylation correlations are enriched in signaling and metabolic pathways. The common denominator across these methylation-regulated genes is the density in MIR retrotransposons of their introns. Moreover, a discrete number of CpGs overlapping enhancers are responsible for regulating most of these genes. Established mouse models recapitulate the dependency of MIR-rich genes on the balanced expression of epigenetic modifiers, while projection of leukemic profiles onto normal hematopoiesis ones further consolidates the dependencies of methylation-regulated genes on MIRs. Collectively, MIR elements on genes and enhancers are susceptible to changes in DNA methylation activity and explain the cooperativity of proteins in this pathway in normal and malignant hematopoiesis.

## Abstract (web summary)

The links between DNA methylation and gene expression are poorly understood on large-scale. Here the authors show that MIR retrotransposons within introns can play this role in acute myeloid leukemia harbouring mutations in epigenetic modifiers.

## Introduction

Mutations in epigenetic modifiers, and specifically in the DNA methylation pathway, are frequently seen in acute myeloid leukemia (AML), resulting in epigenetic deregulation of key biological pathways. DNA methyltransferase 3A (*DNMT3A*) and ten-eleven translocation methylcytosine dioxygenase 2 (*TET2*) are mutated in ~20% and ~10% of AML cases, respectively^1^. In addition, missense mutations in isocitrate dehydrogenase 1 and 2 (herein *IDH1/2*) are seen in about 20% of AMLs. These mutations, which are mostly mutually exclusive with *TET2* mutations, result in the production of the oncometabolite 2-hydroxyglutarate (2-HG), which functions as a competitive inhibitor of TET proteins^1–4^. Previous studies have comprehensively studied the specific methylation profiles associated with AML cases carrying different mutations; while mutations in *DNMT3A* may lead to DNA hypomethylation, those in *IDH1/2* or *TET2* result in different degrees of DNA hypermethylation due to loss of the demethylating function of TET proteins or just TET2, respectively^1,2,5^.

In total, 10–15% of AML patients carry both *DNMT3A* and either *TET2 or IDH1/2* mutations (herein, double mutants)^1,5–7^ and exhibit a methylation profile intermediate to that of either single mutant^5^. In addition, *DNMT3A*-*IDH1/2* double mutants uniquely present with upregulation of KRAS signaling and apoptosis-associated genes, and downregulation of MYC targets compared to normal controls^5^. Murine models of Dnmt3a knock-out (KO) or Idh2 R140Q knock-in mutations recapitulate the epigenetic and expression signatures observed in human cases^5–7^. Phenotypically, double mutant mice exhibit augmented hematopoietic stem cell dysfunction and an increased potential for development of myeloid malignancy^6^. However, the mechanisms underlying this interplay and more specifically, how the resulting changes in DNA methylation leads to subtype-specific transcriptional programs, remain unclear.

More than 50% of the human genome is repetitive and repeat elements are non-randomly distributed in the genome. Pluripotency and proliferation are characterized by the expression of short genes with introns that are enriched in primate-specific Alu elements and Mammalian interspersed repeats (MIRs)^8,9^. By contrast, differentiation and tissue specification genes are depleted from Alu elements but can be enriched in MIR elements^9^. Retrotransposons within genes can regulate splicing or the recruitment of DNA-binding proteins^10,11^. In the hematopoietic system, MIRs can act as insulators^12^ while Alu elements are enriched in topologically associating domains (TAD) boundaries^13^. Finally, retrotransposons may also serve as sites for epigenetic regulation^11,14^.

Here, we show that repeat elements within genes may help explain the relationship between DNA methylation and expression in the context of mutations in the DNA methylation pathway. We carry an integrative analysis of transcriptomic and epigenomic data from our previously published^5^ and the TCGA^1^ cohorts. We find a prominent role of MIR retrotransposons in introns that could act as the link between these mutations and alterations in gene expression programs in normal and malignant hematopoiesis.

## Results

### DNA methylation status of a small set of CpGs within specific TADs is correlated with expression of diverse proximal and distal genes in human *DNMT3A-* or *IDH1/2*-mutant AML

**Fig. 1:**
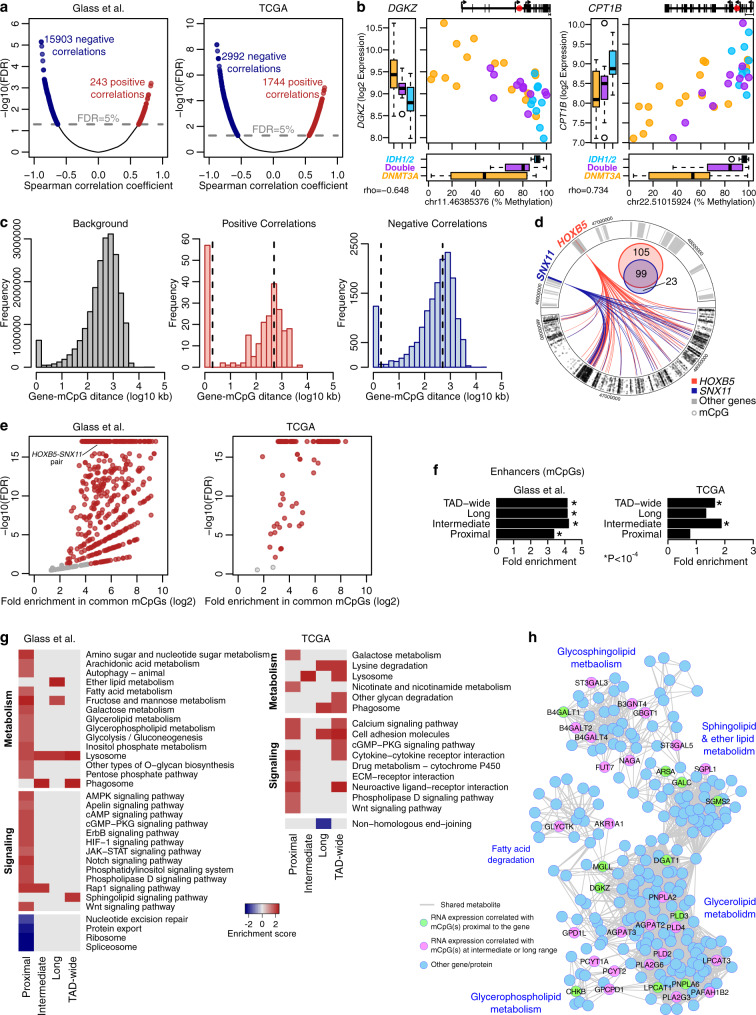
DNA methylation as a regulator of gene expression in signaling and metabolic genes. **a** Volcano plots showing the statistical significance of each mCpG-mRNA correlation vs. the correlation coefficient. **b** Examples of expression-methylation correlations. The graph at the top-right corner depicts the position of the mCpG with respect to the gene locus. The 5’-most as well as the closest transcriptional start sites downstream and upstream to the mCpG are noted. Box plots show the median (center), 25–75 percentile (box), and 5–95 percentile (whisker) from *n* = 16, 9 and 11 *DNMT3A*, *IDH1/2* and double mutant samples, respectively. **c** Histograms of the distance between the mCpG and the gene for all pairs tested (left) and for the significant positive (middle) and negative (right) correlations in the Glass et al. cohort. Note the differences in the scales of the *Y* axes. Dashed lines indicate the distance cutoffs for classifying correlations. Asterisks indicate significant enrichments in the positive (*n* = 243; *p* value < 10^−5^) or negative (*n* = 15,904; *p* value < 10^−5^) proximal correlations (one-sided Chi-squared test). **d** Circus plot of the TAD containing the *HOXB* cluster illustrating the correlations of *HOXB5* and of *SNX11* with mCpGs in the Glass et al. cohort. The two tracks are symmetric; the top visualizes the genes and the bottom the mCpGs. The Venn diagram shows the number of mCpGs correlated with each gene. **e** Scatter plot of fold enrichment in the mCpGs commonly correlated with two genes against the respective FDR (one-sided Hypergeometric test). Each dot represents a gene pair, e.g., *HOXB5* and *SNX11*. **f** Bar plot showing the enrichment of enhancers in the mCpGs with significant correlations (*n* = 10,959 for Glass et al. and *n* = 3549 for TCGA). Asterisks indicate *p* value < 10^−4^ (one-sided Hypergeometric test). **g** Pathways significantly enriched or depleted in the gene list ranked by correlation strength per GSEA. The full list of pathways is included in Supplementary Data 3. **h** Visualization of lipid metabolism genes with significant expression-methylation correlations. Two genes are connected if they use or produce the same metabolite. Genes are colored based on the distance bin with green prevailing purple. Source data are provided as a Source Data file.

We first explored the interplay between DNA methylation and gene expression in AMLs with *DNMT3A* or *IDH1/2* mutations. Given the current understanding of 3D genome organization as well as the well-recognized fact that correlation between cytosine methylation status and expression of the nearest genes is for the most part weak, especially when considering non-CpG island methylation^15^, we performed this analysis by focusing on methylation status within TADs. TADs constitute physical and functional units within the genome, characterized by a high probability of intra-regional interactions that bring into proximity distal regions to exert functional regulation. For this purpose, we performed an unbiased correlation analysis between the methylation status of individual CpGs (mCpGs) and expression levels of genes within the same TAD. Given that the major biological variation in TADs and TAD boundaries at the mega-base scale stems from differences in cell type^16,17^ and that although malignant transformation is accompanied by extensive intra-TAD rearrangements, TAD boundaries at the mega-base scale are largely constant^18^, we performed HiC analysis of primary human CD34^+^ stem and progenitor cells to first define TAD boundaries in these cells (average TAD size = 1.4 Mb; Supplementary Data 1), followed by a correlation analysis of the mCpG status and gene expression levels within these boundaries. Two patient cohorts were included, our previously published cohort^5^ (herein the Glass et al. cohort) and the TCGA^1^. The two cohorts use different technologies to capture DNA methylation that resulted in different representations of the CpG dinucleotides (Supplementary Fig. 1a). We found a total 10,959 and 3549 mCpGs with significant correlations (absolute rho > 0.5; FDR <  5%) to 2566 and 1138 genes in the Glass et al. and the TCGA cohorts, respectively (Fig. 1a and Supplementary Data 2). The majority (98.5% in Glass et al.; 63% in TCGA) of these correlations were negative but with notable exceptions, like the expression of carnitine palmitoyltransferase 1B (*CPT1B*) Notably, across these correlations, double mutant cases fall within an intermediate expression-methylation range compared to the single mutants (Fig. 1b). Next, we split the significant correlations into three groups based on the distance between the gene and the mCpG: proximal (≤2 kb), intermediate (>2 kb and ≤500 kb) or long-range (>500 kb) correlations. We found that both positive and negative correlations were enriched in proximal gene-mCpG pairs (Fig. 1c and Supplementary Fig. 1b). Intermediate and long-range correlations were more likely to include shared genes than with the proximal correlations (Supplementary Fig. 1c). Of note, the number of significant correlations for a given gene did not correlate with the number of tested mCpGs for that gene (Supplementary Fig. 1d).

*HOXB5* and the TAD containing the *HOXB* cluster stood out due to its high number of significant correlations. In the Glass et al. cohort, the expression of *SNX11* within this TAD was correlated with mCpGs that were also significantly correlated with *HOXB5* expression, despite the two genes being 468 kb apart (Fig. 1d). Further genome-wide analyses showed that on average 1% of tested mCpGs could explain the expression of an average of 36% of genes within a given TAD. These overlaps prompted us to examine whether expression of methylation-regulated genes within a given TAD may be explained by a restricted subset of mCpGs within the TAD. Analysis of all genes in a pair-wise manner showed that the same subset of mCpGs is correlated with different genes in both the Glass et al. and the TCGA cohorts, a finding that could not be explained by the genes belonging to the same biological pathway (Fig. 1e and Supplementary Data 3). Notably, in both cohorts, the mCpGs with significant correlations were significantly enriched in enhancers, underscoring a role for DNA methylation regulation of these key regulatory elements (Fig. 1f).

Examining the molecular pathways of genes represented in these correlations, we identified a strong enrichment in signaling and metabolic pathways, including NOTCH, WNT and ERBB signaling, fructose and mannose metabolism, amino and nucleotide sugar metabolism, glycolysis/gluconeogenesis, and lipid metabolism pathways, such as glycerophospholipid metabolism, which included genes like *DGKZ* (Fig. 1g and Supplementary Data 3). These enrichments were strongest in genes with proximal correlations. Network analysis illustrates the extent of the regulation identified through the correlation analysis (Fig. 1h).

Spliceosome, ribosome, protein export and nucleotide excision repair were identified as depleted from genes with high correlation to mCpGs (Fig. 1g). We sought to validate this finding by exploring the 1000 genes with the weakest expression-methylation correlations within each distance bin (herein, W sets). These genes with the weakest correlations were enriched in ribosomal genes and oxidative phosphorylation within the proximal but not in the intermediate or long-range sets (Supplementary Data 3), which is suggestive of a stem cell signature^9^.

Next, we compared methylation of individual mCpGs to methylation at the level of CpG islands (CpGi) by examining the correlation of gene expression with the mean methylation level across all CpG sites in an individual CpGi (Supplementary Data 2). Eighty-nine percent of CpGi with significant correlations contained at least one mCpG with a significant correlation (Supplementary Fig. 1e). As expected given this overlap, there was also significant overlap between genes correlated with CpGi methylation and genes correlated with individual mCpGs resulting in the same biological pathways being identified (Supplementary Fig. 1f, g and Supplementary Data 3).

To determine whether the high methylation-expression correlation of these genes was specific to AMLs with *IDH1/2* or *DNMT3A* mutations, we performed correlation analysis on the same gene-mCpG pairs in AML samples without mutations in *IDH1/2* or *DNMT3A*. We compared the correlation coefficients of these gene-mCpG pairs in the *DNMT3A/IDH1/2* samples with the ones in the WT/WT samples and found that these coefficients were significantly lower in the WT/WT samples in both cohorts (*p* value < 10^−4^; Supplementary Fig. 1h).

Collectively, these results show that in the context of *DNMT3A* and *IDH1/2* mutations, the methylation status of a subset of CpGs is associated with the expression of multiple proximal and distal genes within any given TAD. Moreover, our findings implicate DNA methylation in the regulation of expression of specific signaling, lipid and carbohydrate metabolism genes in these AMLs while other genes and pathways are independent of DNA methylation status.

### Genes with coupled methylation and expression are GC rich, evolutionary neutral and dense in MIR retrotransposons

**Fig. 2:**
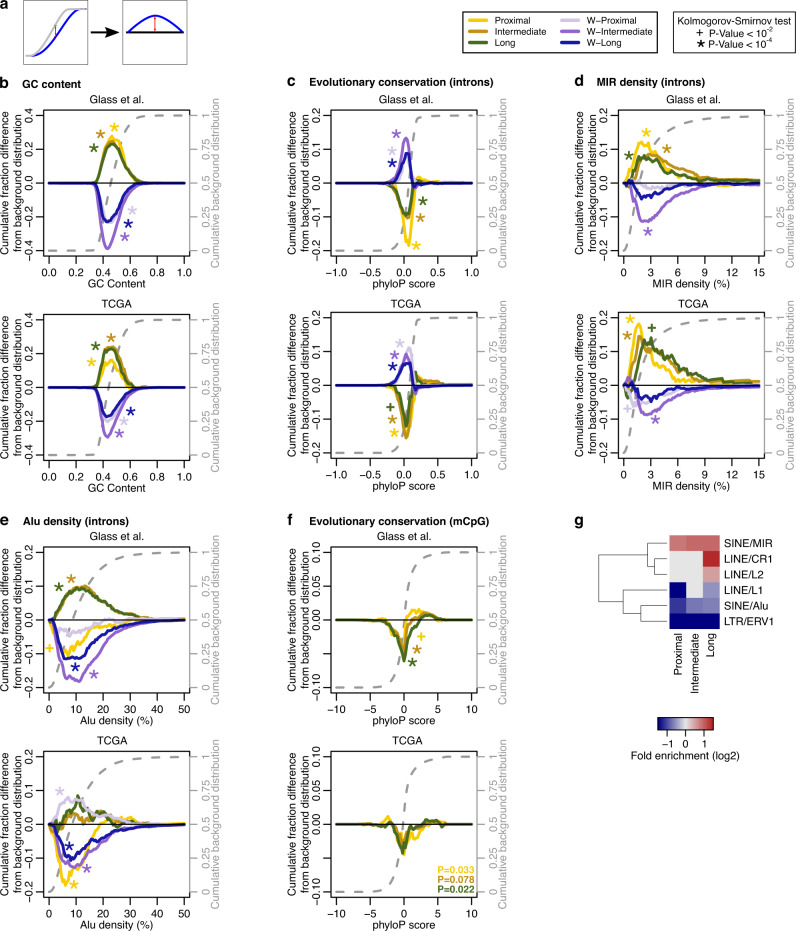
Antithetical architecture of introns of genes with coupled vs. uncoupled expression-methylation. **a** Description of the information presented on the plots. Specifically, plots show the difference between the cumulative distribution of the background genes (dashed gray line) and the cumulative distribution of the respective gene set. **b**–**e** Architectural parameters of the introns of the genes with significant expression-methylation correlations at proximal (*n* = 413 for Glass et al. and 347 for TCGA), intermediate (*n* = 1749 and 680) or long range (*n* = 1344 and 478), and of the 1000 genes with the weakest expression-methylation correlations, i.e., the W gene sets. Plots show the difference between the cumulative distribution of the background genes (dashed gray line) and the cumulative distribution of the respective gene set. Positive values mean a shift of the distribution toward higher values while negative values indicate a shift of the distribution toward lower values. Plots depict analyses of GC content (**b**), evolutionary conservation (**c**) and repetitive element densities of MIR (**d**) and Alu (**e**) elements, for the genes with non-zero respective densities. **f** Evolutionary conservation of the mCpGs that are correlated with genes at proximal, intermediate, or long range. **g** Heatmap illustrating fold enrichment of the mCpGs in repetitive elements. For **b**–**f** asterisks and crosses indicate statistical significance at a *p* value threshold of 10^−4^ and 10^−2^, respectively, per Kolmogorov–Smirnov tests (two-sided). For **f**, hypergeometric tests (one-sided) were used and a *p* value threshold of 2 × 10^−2^. All *p* values are listed in Supplementary Data 4. Source data are provided as a Source Data file.

As genes in signaling pathways and ribosomal genes exhibit specific architectural properties^9,19^, we hypothesized that genes with significant expression-methylation correlations would have biases in their architecture. To test this, we performed an analysis of the architecture and density of repeat elements in our gene sets. Genes with intermediate and long-range correlations, but not those in the proximal set, had shorter introns and an overall higher exonic content (*p* value < 10^−4^ for both cohorts; Kolmogorov–Smirnov test; Supplementary Data 4). However, in both cohorts, all three gene sets had introns and exons that were GC richer and evolutionary more neutral as compared to the background gene population. On the other hand, genes in the W sets of both cohorts had the opposite architecture i.e., were GC poorer and evolutionary more conserved than expected (*p* value < 10^−4^; Kolmogorov–Smirnov test; Fig. 2a–c).

Analysis of retrotransposon content showed that introns, but not exons, of all gene sets with significant correlations were enriched in MIR elements (*p* value < 10^−4^ for both cohorts; Kolmogorov–Smirnov test). On the other hand, the W sets showed no specific biases or were even depleted from MIRs (Fig. 2d). We next examined Alu elements and found that genes with proximal correlations were depleted from Alu elements in both cohorts. Genes with intermediate and long-range correlations were enriched in Alus, in the case of Glass et al., but showed no significant biases in TCGA, though this difference may be the result of the different platforms used to capture methylation status (Fig. 2e).

Next, we examined the properties of those mCpGs that correlate strongly with gene expression. Compared to all mCpGs tested, those that are strongly correlated were significantly less evolutionary conserved, independently of whether they were part of proximal, intermediate or long-range correlations (Fig. 2f). In addition, they were more likely to be part of MIR elements and less likely to overlap Alu or L1 elements in the Glass et al. cohort (Fig. 2g).

Collectively, these findings demonstrate that architectural properties of genes and mCpGs play a role in driving the coupling of expression and methylation in the context of *IDH1/2* and *DNMT3A* mutations, independently of the molecular pathways they are involved in.

### The presence of MIR elements alone is insufficient to explain the complexity of epigenetic regulation

Given the characteristic enrichment of MIR elements in genes with high expression-methylation correlations, we explored whether intronic MIR density alone can explain this. Thus, we first examined the methylation dependency of expression of MIR-dense genes with respect to that of genes with comparable CpG density but with no MIR elements (MIR-zero). We found that only with the proximal setting, genes with the highest MIR density exhibited significantly higher methylation dependency than the MIR-zero genes (Supplementary Fig. 2a).

We then investigated the properties of the specific MIRs that overlap genes and mCpGs with significant correlations in more detail. Previous research has shown that MIR elements can act as insulators on the human genome; these MIRs have an intact B-box by definition and tend to be closer than expected to TAD boundaries^12^. We examined whether the MIRs overlapping the mCpGs with significant correlations can also serve as insulators. However, these MIRs did not show enrichment in B-boxes and their distance to the closest TAD boundary was longer than expected (Supplementary Fig. 2b, c). In addition, when we intersected the known MIR insulators with the MIRs overlapping mCpGs with significant correlations, we found no statistical enrichments or depletions (Supplementary Fig. 2d).

Next, we asked whether the MIR elements enriched in our gene sets with high expression-methylation correlations had expanded during a specific evolutionary window. To test this, we analyzed the divergence scores from the consensus sequence as an approximate for evolutionary age^20^. We found that the MIRs overlapping the mCpGs had no significant biases in terms of divergence scores (Supplementary Fig. 2e) nor were they enriched in any specific MIR subfamilies (Supplementary Fig. 2f).

Collectively, these data suggest that the MIR-driven epigenetic regulation that our correlations reveal is a more general property and not attributed to a specific subset of MIR elements defined by their sequence. Furthermore, our data indicate that the contribution of MIR elements to epigenetic regulation of gene expression in AML is more complex than simple MIR density or intronic sequence information alone, and that an additional level of regulation must be involved.

### DNA-binding protein complexes recruited to MIR elements can bridge mCpGs with the correlated genes

Given that sequence information alone was insufficient to explain the correlation between expression and methylation, we hypothesized that an additional layer of regulation played a role in linking the identified genes and mCpGs. Therefore, we next incorporated information from ChIP-seq profiles of hematopoiesis- and leukemia-relevant DNA-binding proteins and multiprotein complexes (DBP/Cs)^21,22^ to explore whether specific DBP/Cs can bridge the mCpG with the gene, serving as a bridge for epigenetic regulation of gene expression. We further hypothesized that since MIR elements are enriched at both genes and mCpGs involved in these correlations, they may play a role in facilitating this bridging function of DBP/Cs.

**Fig. 3:**
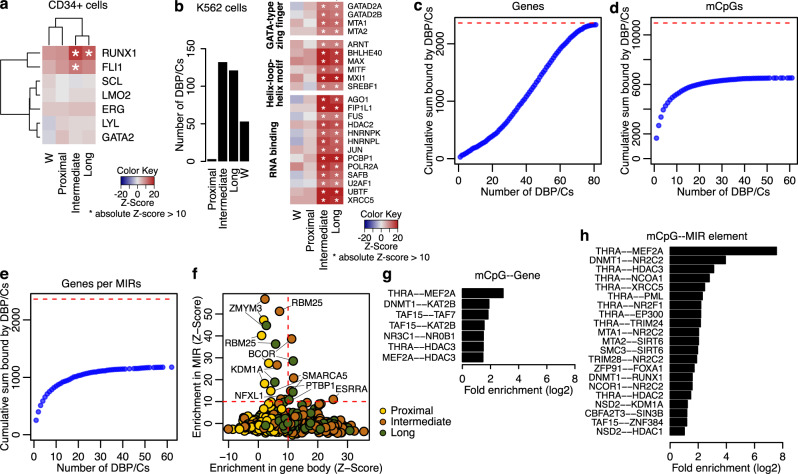
Enrichment of DNA-binding proteins and multiprotein complexes (DBP/Cs) in genes with intermediate- and long-range correlations. **a** Heatmaps showing the enrichment/depletion of transcription factor binding in CD34^+^ cells. The value represents the *Z*-score of the observed overlap of ChIP-seq peaks with reference to a simulated expected distribution (see Methods for details). Asterisks indicate an absolute *Z*-score greater than 10. **b** Bar plot showing the number of DBP/Cs from ENCODE significantly enriched (*Z*-score >10) in each gene set. Heatmap showing the enrichment/depletion of selected DBP/Cs from ENCODE enriched in the genes with intermediate- and long-range correlations but not in the ones with proximal correlations. Asterisks and notations are same as in **a**. **c**, **d** Plots showing the number of genes (**c**), mCpGs (**d**) or genes containing at least one MIR element (**e**) that are bound by up to N transcription factors (*x* axis). This is shown in a cumulative manner, e.g., up to 20 DBP/Cs bind a total of 393 unique genes with significant intermediate or long-range correlations (**c**). Red dashed lines show the number of all respective genes or mCpGs with significant correlations. **f** Plot showing the *Z*-scores of ENCODE’s DBP/Cs (same as in **b**) in gene space (*X* axis) against the respective in MIR space (*Y* axis). Red lines demarcate the significant thresholds. The same protein can be seen twice as it refers to the analysis based on different gene sets (marked by different colors). **g**, **h** Bar plots showing the enrichment of the interactions between DBP/Cs in the mCpG-gene pairs with significant methylation-expression correlations (*n* = 16,142). The first protein binds the mCpG and the second binds at the gene (**g**) or a MIR element within the intronic space of the gene (**h**). For both plots, *p* values were calculated with a hypergeometric test (one-sided), corrected to FDR and we show the significant results, FDR < 5%. All *Z*-scores and DBP/C interaction pairs are included in Supplementary Data 5. Source data are provided as a Source Data file.

To statistically evaluate enrichment of specific DBP/Cs in our gene sets, we performed Monte–Carlo simulations by shuffling these gene regions within the respective genomic space; thus, we approximated a distribution of an expected number of DBP/C binding sites and compared it with the number of peaks or sites at hand (see Methods; Supplementary Data 5). By using ChIP-seq data from 8 hematopoietic transcription factors in CD34^+^ cells^21^, we found that RUNX1 is enriched at genes from all gene sets but is only highly enriched in genes with intermediate- and long-range correlations. Similar observations could be made for FLI1, whose binding was enriched only in genes with intermediate-range correlations (Fig. 3a). We further investigated the overlap of our gene sets with DBP/C binding sites in K562 cells from ENCODE^22^. In this dataset, genes with intermediate and long-range correlations were enriched in 132 and 121 DBP/Cs, respectively; genes with proximal correlations were enriched only in 4 DBP/Cs while the W set was enriched in 53 (Fig. 3b). Once again, RUNX1 was found enriched in genes with intermediate and long-range correlations but not in the proximal or W sets. There was no DBP/C uniquely enriched in the genes with proximal correlations, but we counted 81 DBP/Cs enriched in genes with intermediate or long-range correlations but not in the W set (Supplementary Data 5). Among these 81 proteins, the three largest groups of proteins corresponded to GATA-type zinc finger proteins, helix-loop-helix transcription factors and RNA-binding proteins (Fig. 3b).

We focused on the genes with significant intermediate or long-range correlations (henceforth, the genes with distal correlations, or the D gene set) and the 81 DBP/Cs uniquely enriched in them. All 81 DBP/Cs have peaks in an average of 1187 genes with distal correlations, covering 2332 genes (out of the 2363; 99%) (Fig. 3c). Each of these 81 DBP/Cs also binds an average of 545 mCpGs with significant correlations, covering a total of 6521 mCpGs, i.e., 60% of the mCpGs with significant correlations (Fig. 3d). Examining the binding of these 81 DPB/Cs on MIR elements, we identified 1177 genes (50% of the D genes) that contain at least one MIR element bound by at least one of these 81 DBP/Cs, covering a total of 2669 MIRs (Fig. 3e).

To statistically evaluate the overlap between DBP/Cs with MIRs, we performed Monte–Carlo simulations as above but restricted the genomic areas of interests to the MIR elements overlapping the respective gene sets. We found eight DBP/Cs with significant overlap with MIR elements embedded in genes with significant expression-methylation correlations, four of which were also enriched in the analysis at the gene level, namely BCOR, ESRRA, PTBP1 and SMARCA5 (Fig. 3f).

We then examined the potential bridging of mCpGs with genes by two DBP/Cs that physically interact, as previously done in similar contexts^23,24^. Specifically, we used a human protein-protein interaction (PPI)^25^ network to identify interactions between the DBP/Cs and identified all PPIs where one protein binds to the mCpG and the other to the gene (see Methods). We first asked whether there are PPIs specifically enriched in the significant correlations as compared to the background (i.e., all possible PPI pairs in the correlated mCpG-gene pairs). We identified seven pairs significantly enriched in the correlations, with the most significant being the interaction between THRA binding at mCpGs with MEF2A binding at genes (Fig. 3g and Supplementary Data 5). To evaluate the role of MIRs, we repeated this analysis but only focused on PPIs where the second DBP/C binds a MIR embedded in the intronic space of the gene. We found 21 protein pairs that were significantly enriched in the correlations. As above, the top pair was THRA-MEF2A, with a fold enrichment that was two orders of magnitude larger than the respective enrichment at the gene level. THRA was found bound at the mCpG in an additional seven significant PPIs. Notably, many of these PPI pairs included at least one epigenetic modifier, including EP300, HDAC1, HDAC2, HDAC3, KDM1A, NSD2, DNMT1 and SIRT6 (Fig. 3h).

Given that MIR elements can be sites of epigenetic regulation themselves, we hypothesized that the MIRs overlapping genes or mCpGs with significant correlations would also exhibit biases in regulatory features. Using histone modification profiles from lineage^-^CD34^+^CD38^−^ cells^26^, we found no enrichment in histone marks in the MIRs overlapping genes but significant enrichment of H3K27ac and H3K4me1 in the MIRs overlapping the mCpGs (Supplementary Fig. 3), in agreement with the overlap with enhancers described above (Fig. 1f).

Our results show that the genes with long-range coupled expression-methylation within a TAD are enriched in DBP/Cs, including RUNX1, FLI1 and GATA-type zinc finger proteins. Such proteins, by themselves or as part of large protein complexes, particularly those including proteins like BCOR and PTBP1, seem to be recruited to the MIR elements embedded in the introns of these genes. These DBP/Cs may act as the bridge between the gene and the respective mCpGs, as they also have ChIP-seq peaks on almost a third of the mCpGs with significant correlations. We were also able to identify potential bridges facilitated by the physical interaction of two DBP/Cs and in this model, MIRs can explain part of these DBP/C pairs. Collectively, our findings argue that MIR retrotransposons can facilitate the epigenetic regulation of gene expression as observed in these AML subtypes by recruiting specific DNA-binding proteins.

### Retrotransposons overlapping protein-coding genes and enhancers are differentially methylated among human AML subtypes

**Fig. 4:**
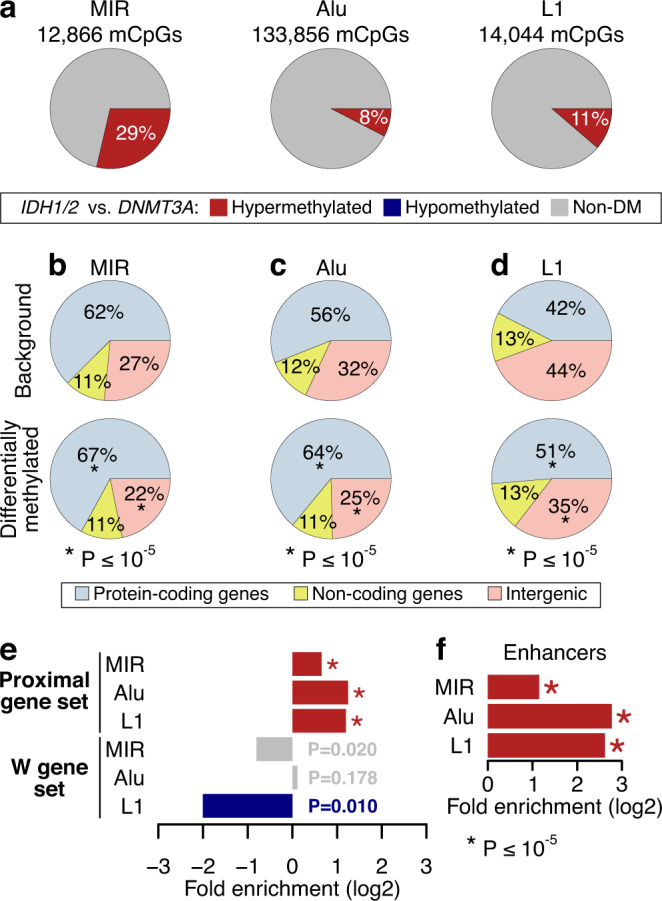
DNA methylation changes on MIR, Alu and L1 elements. **a** Pie charts showing the percentage of differentially methylated cytosines (DMCs) overlapping the respective elements. **b**–**d** Pie charts showing the enrichment of protein-coding genes around the DMCs with reference to all cytosines included in the analysis, statistically evaluated with chi-squared tests. **e** Fold enrichment of the proximal or W gene sets overlapping the DMCs of each repetitive element. **f** Fold enrichment in enhancers overlapping the DMCs of each repetitive element. For **e**, **f**, *p* values are noted on the figures, asterisks indicate enrichment or depletion with *p* value <10^−5^ per hypergeometric test (one-sided) for *n* = 3685, *n* = 10,162 and *n* = 1585 DMCs overlapping MIR, Alu or L1 elements, respectively. Source data are provided as a Source Data file.

Given the differences in genomic architecture of mCpGs with high methylation-expression correlations, we hypothesized that the methylation status of retrotransposons may be differentially methylated among AML subtypes. To test this, we first identified differentially methylated mCpGs (DMCs) between *IDH1/2*- and *DNMT3A*-mutant AMLs, since they represented the two extremes of our correlation analysis, and then focused on cytosines that overlap MIR, Alu and L1 elements. MIR elements were more likely to be differentially methylated and they were 100% hypermethylated in the *IDH1/2* subtype (Fig. 4a and Supplementary Data 2). Next, we looked at the relationship of these repeat element DMCs to both coding and non-coding genes and observed that these DMCs were more likely to be annotated to protein-coding genes (*p* value < 10^−5^ for all three families of repeats; chi-squared test; Fig. 4b–d). Notably, DMCs at MIR, Alu and L1 elements were enriched in the proximal gene set (*p* value < 0.01; hypergeometric test) but not significantly overlapping with the negative control W gene set, pointing to a role of these retroelements in establishing relevant methylation-expression correlations. L1 elements were an exception, the DMCs of which were significantly depleted from the W genes (Fig. 4e). We further intersected the DMCs with active and poised enhancer regions identified in hematopoietic stem and progenitor cells^26^ and found that the DMCs of all three families were significantly enriched at enhancers (*p* value < 10^−5^; hypergeometric test; Fig. 4f).

These results further argue that retrotransposons are sites of epigenetic regulation and indicative of AML subtype differences, particularly those retrotransposons overlapping coding genes and/or enhancers with a methylation-expression coupling.

### Mutations in Dnmt3a or Idh2 affect the expression of genes with specific retrotransposon content in mouse hematopoietic cells

Given the evolutionary concordance of MIR and Alu/B1 elements in the human and murine genomes^27^, we took advantage of murine models of *Dnmt3a* and *Idh2* to test our hypothesis that mutations in these genes would directly affect MIR-dense genes. We mined the gene expression data for Dnmt3a KO, Idh2^R140Q^ mutant, and double-mutant samples, generated by Zhang et al.^6^ to explore the interaction of the two mutations in a context with no additional confounding mutations^28^.

**Fig. 5:**
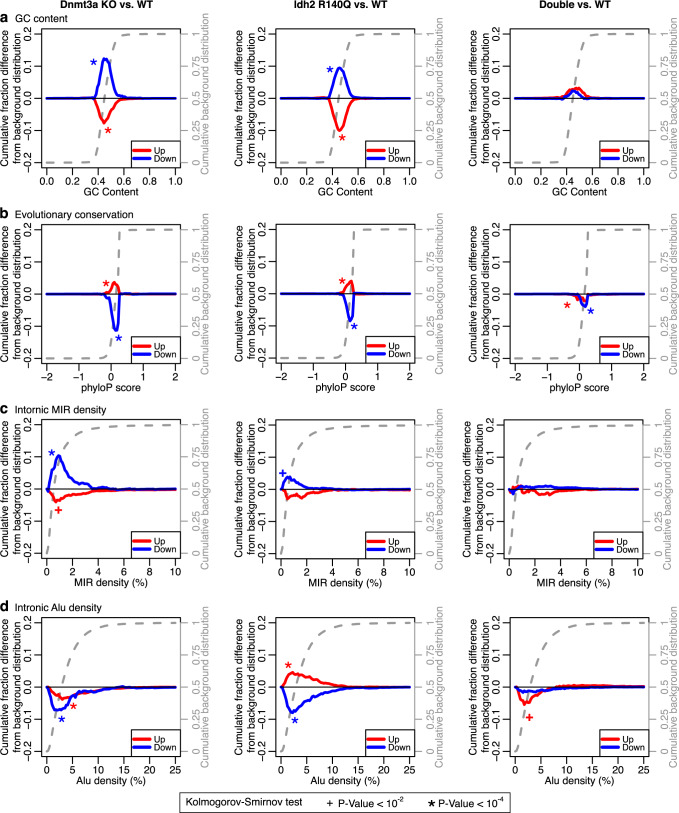
Mouse models recapitulate the dependency of MIR-rich genes on proper Dnmt3a and/or Idh2 function. Difference between the cumulative distribution of the background genes (dashed gray line) and the cumulative distribution of the up- or downregulated genes after Dnmt3a KO (left column), Idh2 R140Q mutation (middle column) or both Dnmt3a KO and Idh2 R140Q (right column) in GC content (**a**), evolutionary conservation (**b**), MIR density (**c**) and Alu density (**d**). Positive values mean a shift of the distribution toward higher values while negative values indicate a shift of the distribution toward lower values. Asterisk and crosses indicate statistical significance at a *p* value threshold of 10^−4^ and 10^−2^, respectively, per Kolmogorov–Smirnov tests (two-sided). All *p* values are listed on Supplementary Data 4. Source data are provided as a Source Data file.

We first examined the differentially expressed (DE) genes between single mutants and control samples. Genes significantly downregulated were enriched in signaling pathways and lipid metabolism genes, while ribosomal genes were enriched in the upregulated set (Supplementary Data 2 and 3). Introns of downregulated genes in both single mutant genotypes were GC richer and evolutionary more neutral than expected, while introns of upregulated genes were GC poorer and evolutionary more conserved (Fig. 5a, b and Supplementary Data 4). Meanwhile, introns of downregulated genes in both genotypes were also denser in MIRs and sparser in Alu elements (Fig. 5c, d). Analysis of genes DE between double mutants and control samples revealed that downregulated genes were enriched in cell cycle and DNA replication pathways (Supplementary Data 3). Notably, introns of these downregulated genes had no significant differences in GC content and MIR and Alu densities compared to control samples while evolutionary conservation, was marginally significantly different (Fig. 5a–d). With the exception of Alu density, similar observations could be made for genes upregulated in the double mutants.

The above results argue for the importance of a common architecture and retrotransposon content of the genes affected by single mutations in *Idh2* or *Dnmt3a*. The observed reversal of architectural biases in genes DE between the double mutants as compared to either single mutant suggests the dependency on a balance between methylation and demethylation of the genes with this architecture.

### AML subtypes align with expression trajectories of normal human hematopoiesis that are biased for evolutionary conservation and GC and retrotransposon content

**Fig. 6:**
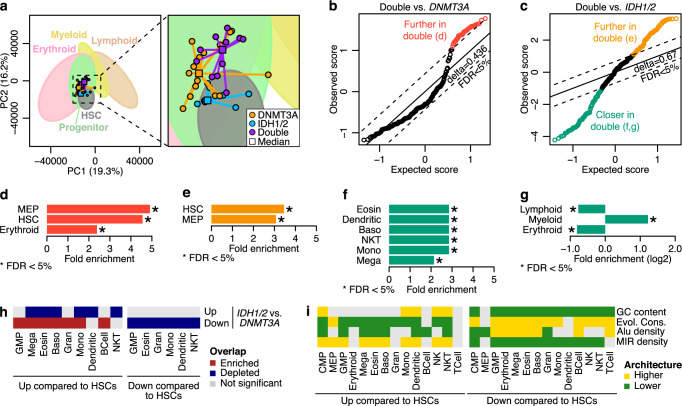
AML subtype differences can be projected on MIR-biased expression trajectories of normal hematopoiesis. **a** PCA plot of the ranked-normalized normal and AML expression profiles. For simplicity, only AML samples are plotted, while ellipses are fitted to show the space occupied by each lineage. For each AML subtype, the median projection per principal component was calculated and plotted as a square. The exact coordinates of each sample are included in Supplementary Data 6. Significance Analysis of Microarrays (SAM) plots showing the statistically significant differences in the distances between Double and *DNMT3A* (**b**) or *IDH* mutant samples (**c**). Each circle represents a normal sample. If the difference between the observed score and the expected, as calculated after random permutations, is larger than the threshold delta corresponding to an FDR of 5%, then the distance of the *DNMT3A* mutant samples (**b**) or the *IDH* mutant samples (**c**) is significantly different than the distance of the double mutants. Samples that exceed this threshold are colored orange, green or yellow. **d**–**g** Enrichment in cell types on the samples significantly closer (*n* = 43 samples and *n* = 61 samples for *DNMT3A* or *IDH1/2* mutants, respectively) or further (*n* = 74 samples for *IDH1/2* mutants) to the double mutants as compared to the single mutants. Colors match the respective groups of **b**, **c**. Asterisks indicate statistical significance per hypergeometric test (one-sided). **h** Heatmap showing the overlap of differentially expressed genes between *IDH1/2* and *DNMT3A* AML mutants with the DE genes across normal hematopoiesis. **i** Heatmap showing the architectural biases in the differentially expressed gene of each cell type as compared to HSCs in normal human hematopoiesis. Baso basophils, CMP common myeloid progenitor cells, Eosin eosinophils, GMP granulocyte-monocyte progenitor cells, Gran granulocytes, HSC hematopoietic stem cell, Mega megakaryocytes, MEP megakaryocyte-erythroid progenitor cells, Mono monocytes, NK natural killer cells, NKT NK T cells. Source data are provided as a Source Data file.

We hypothesized that the AML expression programs as we captured through the correlations contain an underlying differentiation-specific component. To test this, we aligned the AML expression data along normal hematopoiesis expression trajectories. Using publicly available expression profiles obtained from different stages of human hematopoiesis^29^, we projected the leukemic samples onto the PCA space defined by the normal samples (Fig. 6a). To statistically evaluate these projections, we searched for significantly different distances amongst each AML subtype along different stages of normal hematopoiesis, in a fashion similar to examining for DE genes. We identified 44 and 62 normal cell types that were more distant to double mutants as compared to *DNMT3A*-mutant and *IDH1/2*-mutant samples, respectively (Fig. 6b, c and Supplementary Data 6). Normal cells that were furthest from double mutants were enriched in hematopoietic stem cells (HSC) and megakaryocyte-erythroid progenitors (MEP) (Fig. 6d, e). Another 75 normal samples were also found to be closer to double mutants than to *IDH1/2*-mutants (Fig. 6c) and these were enriched in mature myeloid cells (Fig. 6f, g). We next examined the overlap between DE genes amongst AML subtypes and genes DE in progenitor or mature blood cells as compared to HSCs. We found a significant overlap between genes upregulated in *IDH1/2*-mutant AMLs as compared to *DNMT3A-*mutant with genes upregulated during normal differentiation, particularly of the myeloid lineage, confirming the stemlike phenotype previously described for DNMT3A-mutant AMLs^5^ (Fig. 6h).

The above alignments prompted us to examine the architecture of DE genes between the different stages of differentiation as compared to HSCs (Supplementary Data 2 and 4). We observed that, in general, introns of upregulated genes were more GC dense, evolutionary more neutral, MIR-denser and Alu-sparser than expected, largely independent of cell type (Fig. 6i). In contrast, downregulated genes mostly had the opposite architecture.

Collectively, our results argue that leukemic cells can be projected on expression trajectories of normal hematopoiesis that are comprised from expression differences of MIR-dense genes. The interaction of both mutations in double mutant leukemic samples results in these resembling more differentiated cell types than either single mutant, arguing that these leukemias have reached or have originated from a distinct MIR-defined expression state.

## Discussion

Mutations in AML that impact different components of the DNA methylation pathway have been shown to have distinct and opposing effects on gene expression and DNA methylation^5–7,30–34^. However, the underlying effector genomic features driving the interplay between cytosine methylation and expression and resulting in these differences were still unknown. In this study, we performed an unbiased correlation analysis of gene expression and DNA methylation using TAD coordinates to define functional genome compartments. Notably, both genes and mCpGs involved in these significant correlations display distinct genomic architectural features and are dense in MIR retrotransposons, a characteristic that was conserved across species. Moreover, our data point to a potential role of MIR elements in the recruitment of DBP/Cs to help link coding genes and regulatory CpGs. Finally, normal hematopoiesis expression trajectories also involve expression differences in MIR-dense genes. Our results point to a mechanism involving MIR retrotransposons as main substrates or recruiters of DNMT3A and TET2 that regulates normal HSC differentiation and is hijacked by leukemic cells. A core finding of our study is the observation that CpG methylation and gene expression are linked through MIR retrotransposons, both because mCpGs are enriched in MIR retrotransposons and enhancers, but also because the gene bodies of methylation-dependent genes in turn are enriched in MIR elements. This architectural linkage was independent of genomic distance, but longer distances seem to additionally require the bridging by DBP/Cs.

Methylation at CpG dinucleotides or islands is mostly known for its negative effect on gene expression when overlapping promoters, but recent research has shown methylation at enhancer regions is equally, if not better, associated with gene expression^35,36^. By analyzing enhancers from a panel of human cell types, Bell and Vertino found that CpG islands within enhancers are hot spots for genomic contacts and for DBP/C binding as well as less evolutionary conserved than ones in promoters^37^. These observations agree with our findings in the context of AML. Furthermore, enhancers’ CpG methylation patterns are disrupted by *TET2*, *IDH1/2* and *DNMT3A* mutations in leukemia^5,38^. Within this context, our results in *DNMT3A* and *IDH1/2* AMLs describe a gene expression regulatory landscape within TADs driven by epigenetic modifications at enhancer regions that can be disrupted by mutations in epigenetic modifiers.

Previous studies have shown that proliferation and stemness are characterized by increased expression of retrotransposons and short MIR-dense and Alu-dense genes^9^. However, as cells differentiate, they express longer Alu-sparse and occasionally MIR-dense genes^9^. The expression of both coding and non-coding genes can indeed be regulated by retrotransposons^9,11,39,40^ and these repeat elements play key roles beyond being sources of genomic instability and mutations^41–43^. In this study, we build upon those lines of evidence and argue that normal HSC differentiation and AML require the proper utilization of MIR sequences as sites of epigenetic regulation of gene expression. Furthermore, Colombo et al. found that transposable elements, including Alu (MIRs were not analyzed), were suppressed in leukemic stem cells as compared to pre-leukemic stem cells by mechanisms involving post-transcriptional regulation^44^. MIR and Alu elements have also been found enriched in genes correlated with small non-coding RNA levels^45^. Thus, it is intriguing to hypothesize an interplay between epigenetics and non-coding RNAs targeting MIR sequences.

Examining proximal correlations with respect to distal (intermediate and long-range) ones, we observed that although both types contain genes enriched in MIR elements, the proximal correlations contained Alu-sparse genes while the distal ones contained Alu-dense genes. Based on prior observations of MIR and Alu densities in differentiation^9^, we can postulate a synergist role of these two retrotransposons as a mechanism to regulate distal epigenetic-expression links. Indeed, we observed that in normal hematopoiesis the establishment of cell identity through the expression of differentiation and cell-type-specific genes requires the selective expression of genes that are depleted from Alu elements but still enriched in MIR elements. We postulate that their regulation happens through methylation of proximal mCpGs. Thus, proximal correlations may capture the commitment on a specific differentiation trajectory while distal correlations may be indicative of interactions reflective of cellular stemness. However, despite these overall trends, it is likely that a combination of genes regulated through these two types of correlations may be required for normal cell differentiation and during leukemogenesis.

Although MIR elements appear to play a significant role in linking DNA methylation and gene expression, we cannot rule out additional effects of repeat elements’ DNA methylation on AML’s biology. For instance, DMCs overlapping Alu and L1 elements were more likely to be located at enhancers, which could impact processes like chromatin domain formation or enhancer RNA production that are not directly captured by our correlations.

Our study is impacted by the limitations in current technologies, which do not capture the full spectrum of CpG methylation sites. Particularly on repeat elements, the comprehensive evaluation of CpG methylation is a challenging and currently unresolved problem in the field that requires the development of new methodologies. However, we believe that our study provides strong evidence for the need of future studies to evaluate the full spectrum of epigenetic changes at repeat elements in leukemia and likely other malignancies as well. Furthermore, while our results on DBP/Cs as potential bridging mechanisms are tantalizing, we cannot fully evaluate the impact of DNA methylation on changes in the binding affinity of protein complexes on DNA as a mechanism behind the epigenetic-transcriptomic coupling. To evaluate such hypothesis, for each DNA-binding protein analyzed, we would need data on the effect of methylation on its binding affinity, its binding profile in *IDH1/2* and *DNMT3A* single- or double-mutant samples; data that exceed the scope of the current study.

So, how does genome architecture and retrotransposons explain the interaction of mutations in *DNMT3A* and *IDH1/2*? Spurred from evidence on embryonic stem cells, Parry et al. put forward the hypothesis that methylation turnover, i.e., a rapid methylation-demethylation cycle on cytosines catalyzed by DNMT and TET enzymes, is necessary for HSCs exiting from pluripotency and normal hematopoietic differentiation^46^. Mutations in *DNMT3A* or *TET2/IDH1/2* will disrupt the methylation turnover and proper gene expression; however, mutations in both genes may restore the balance and consequently expression of MIR-dense genes. This is observed in our results in both human and mouse data: double mutants fall in an intermediate state, between either single mutants. Thus, MIR retrotransposons may not only act as bookmarks on the genome bridging mCpGs with genes but also serve as the points on the genome at which this methylation-demethylation cycle occurs in normal and malignant hematopoiesis.

## Methods

### Expression and methylation data acquisition, processing and filtering

For the Glass et al. AML cohort^5^, we downloaded the methylation and expression data from Gene Expression Omnibus (GEO), accession numbers GSE98352 and GSE6891^47^, respectively. Specifically, we used the methylation calls directly from GEO and filtered out cytosines with a coverage less than 10 in any of the sample. All cytosines fell in CpG dinucleotide context. For CpG islands, we averaged the methylation levels of the mCpGs that overlapped the island and had the appropriate coverage. The expression datasets for the samples with methylation calls were downloaded as CEL files from GEO, RMA and quantile-normalized and the 50% most expressed protein-coding genes were considered for downstream analyses.

For the TCGA cohort^1^, the level 3 RPKM RNA expression matrix and level 3 DNA methylation matrix were downloaded from the GDC portal (https://gdc.cancer.gov/about-data/publications/laml_2012; accessed on January 15, 2020) and mutation calls were drawn from the respective TCGA publication. Samples with *IDH1*, *IDH2* or *DNMT3A* mutations were considered; those with a co-occurring *TET2* mutations were excluded. The top 50% most expressed genes were kept in downstream analyses. We kept in our analyses methylation probes that had a methylation value of >0.3 in more than 3% of the samples.

For normal human hematopoiesis, we downloaded the CEL files of GSE24759^29^ from GEO, RMA-normalized them and kept the 75% most expressed (sorted by average expression in the whole data matrix; *n* = 9565). For our analyses, we grouped the 211 samples into 15 cell types without distinguishing among stages of the same cell type (e.g., among naïve, effector or memory T cells).

Mouse expression data with accession code GSE60055^6^ were downloaded as sra files from GEO, mapped on the GRCm38 mouse genome using STAR version 2.7.1a and FPKM values were calculated using the *rsem-calculate-expression* command of RSEM version 1.2.28^48,49^. For our analyses, we kept genes with a median expression value of at least 1 FPKM in the analyzed samples.

### Genomic data acquisition and processing

We integrated information from multiple publicly available databases and published datasets. For consistency across the multiple data types, we used the hg19/GRCh37 human genome and gene annotations (exon and intron ranges and gene types) from ENSEMBL. For mouse we used mm10/GRCm38. Repetitive elements for human GRCh37 (version 4.0.5) and mouse GRCm38 (version 4.0.5) were downloaded from RepeatMasker (http://repeatmasker.org/). Divergence scores were defined as the sum of percentages of substitutions, insertions and deletions. B-Boxes were defined as GTTCNANNC and were searched in the genomic segments defined by the MIR elements in sense or antisense orientation. Information on metabolic pathways was downloaded from KEGG^50^. ENCODE DNA-binding protein ChIP-seq profiles^22^ for hg19/GRCh37 were downloaded from UCSC Genome Browser using Table Browser (encRegTfbsClustered primary table; accessed on 20 August 2019) and filtered so that only experiments on K562 cells were kept. Evolutionary conservation data, specifically conservation scoring by phyloP for 45 vertebrate genomes^51^, were also downloaded from the USCS Genome Browser (accessed on 11 October 2019). Enhancer coordinates and histone mark ChIP-seq profiles were drawn from the Adelman et al. study^26^, while ChIP-seq profiles from DBP/Cs were drawn from the Beck et al. study^21^. Coordinates for CpG islands (cpgIslandExt) were downloaded from the UCSC Genome Browser using the Table Browser tool. Genome arithmetic (e.g., region intersection) was performed with bedtools version 2.28.0^52^. Cross-checked human protein-protein interactions normalized at the gene level were downloaded from the PICKLE database version 3.3.

### TAD identification

Mobilized peripheral blood CD34+ cells were purchased from Fred Hutchinson Hematopoietic Cell Procurement Services. Hi-C was performed in replicate on freshly thawed one million CD34+ cells using the Arima-HiC Kit (Arima Genomics, A510008) following manufacturer’s protocol for low input crosslinking and library preparation with Accel-NGS 2S Plus DNA Library Kit (Swift Biosciences, 21024). Libraries were sequenced on a NovaSeq 6000 (Illumina) in paired-end mode by the Sylvester Comprehensive Cancer Center Onco-Genomics Shared Resource. The resulting data were analyzed with Juicer (Version: 1.5.6)^53^ on AWS (https://aws.amazon.com/) with default parameters. The two replicates were analyzed separately. The two .hic files were combined and the *arrowhead* function from Juicer was used for calling TAD regions with 40 K resolution (Supplementary Data 1). Overlapping domains were merged into one consecutive TAD. These TADs were subtracted from the whole genome and the remaining regions were also considered as TADs for the purposes of our correlations. This methodology split the human chromosomes into 3103 TADs.

### Statistical analyses and data visualization

We used hypergeometric test when the statistical question could be formulated as to evaluate over/under-enrichment in a sampling set from a larger finite population without replacement. For instance, to examine whether the mCpGs with significant correlations were more likely to have a specific (discrete) characteristic, like overlap with a ChIP-seq peak for a DNA-binding protein, an enhancer or a MIR element. For this purpose, we counted the number of successes in the selected set (e.g., number of mCpGs with significant correlations that also overlap an enhancer) and compared it to the number of successes in the whole (finite) population (e.g., the number of all mCpGs that overlap enhancers). The hypergeometric test is based on a discrete probability distribution that makes it appropriate for this type of analyses with the null hypothesis being that there is no selection bias from the background population.

We used a one-sample Kolmogorov–Smirnov test when our statistical question could be formulated as to examine whether a continuous variable in the selected set originates from the specific distribution of the background set; with the null hypothesis being that the distribution of the values in the selected does not differ from the background one. For instance, we employed this test when we examined the density of MIR elements in the introns of genes with significant correlations. Our null hypothesis in this case is that the genes selected by the expression-methylation correlations had the same MIR density distribution as the background set, i.e., were chosen randomly. Thus, the Kolmogorov–Smirnov test is appropriate to compare the distribution of the values from the correlation set with respect to the background cumulative distribution. We note that the test is non-parametric and thus does not require the parameters to follow a specific distribution. In addition to retrotransposon content, we also used this test when we tested for biases in GC content and evolutionary conservation.

Spearman correlation coefficients and associated *p* values were computed for each gene and mCpG (in the case of the Glass et al. cohort) or methylation probes (in the case of TCGA data) within the same TAD. Specifically, each gene, whose expression passed the criteria described above, was correlated with all the mCpGs (for the Glass et al. cohort) or methylation probes (for the TCGA cohort) that both overlapped, at least partially, with the same TAD. *p* values were then corrected to FDR values and expression-methylation pairs with an associated FDR < 5% were considered. Based on the location of the mCpG with respect to the gene, i.e., the union of all its exons and introns, correlations were classified as proximal, if the mCpG was within the gene body or within a 2 kb window of the gene, as intermediate, if the mCpG was not proximal to the gene but was within 500 kb and as long if the mCpG further than 500 kb from the gene. DAVID^54^ was run on the genes with significant methylation-expression correlations (i.e., the proximal gene set), with all the genes participating in the correlation analyses serving as the background. As an orthogonal approach, we ranked genes based on the maximum absolute correlation coefficient of all the proximal, intermediate- or long-range mCpGs or methylation probes. This allowed us to perform Gene set enrichment analysis (GSEA)^55^. We also extracted the 1000 genes with the weakest correlations (i.e., the W gene set) at the proximal, intermediate or long-range settings. For both DAVID and GSEA, the Supplementary Data include all results for a threshold up to 10% FDR. We used hypergeometric tests to evaluate the overlap of the mCpGs with significant correlations with poised and active enhancers from the Adelman et al. study^26^, with DBP/C binding sites from Beck et al.^21^ and DBP/C binding sites from ENCODE^22^.

Differential expression analyses for the mouse data were done with Significance Analysis for Microarrays^56^ (SAM; 5000 permutations and 5% FDR threshold) after log2-transforming the data matrices. For the Zhang et al. study (GSE60055), we used the MEP cells for our analyses. For the single mutant genotypes, two replicates were analyzed in the original study; thus, for each gene we computed the average of the two replicates and added it as a third pseudo-replicate.

Differential methylation analysis of retrotransposons was done using the mCpGs that overlap each retrotransposon class separately, with coverage more than 10 and non-zero methylation in more than one of the samples in each class (e.g., *IDH1/2* mutants). As mapping by Bismark discards reads with multiple alignments on the genome with the same number of mismatches^57^, our approach only considered sequencing reads that could be uniquely aligned in sequences that are highly repetitive on the genome. Thus, we could not make assumptions on whether the data follow a specific distribution and therefore used the non-parametric SAM (5000 permutations, FDR threshold of 5%). We used chi-squared tests to examine the distribution of the DMCs in protein-coding genes, non-coding genes or in intergenic space. We also used hypergeometric tests to examine the overlap of DMCs with poised and active enhancers^26^.

All statistical analyses were conducted in python version 2.7.16 or R version 3.6.1. We made use of the *numpy* (v. 1.16.5) and *scipy* (v. 0.19.1) packages in python and *amap* (v.0.8-18), *circlize*^58^ (v. 0.4.12), *data.table* (v. 1.3.16), *dendextend*^59^ (v, 1.14.0), samr (v. 3.0) and *VennDiagram*^60^ (v. 1.6.20) packages in R as well as Cytoscape version 3.7.2^61^.

### Overlap of genes and MIR elements with DNA-binding proteins

To statistically evaluate whether DNA-binding proteins (DBP/C) from Beck et al.^21^ or ENCODE^22^ were more likely to bind on genes with significant correlations, we performed a Monte–Carlo simulation. Each DBP/C was analyzed individually for each set of genes with proximal, intermediate or long-range correlations or for each respective W set of genes. In more detail, we first counted how many genes overlapped with at least one DBP/C peak (to be called “observed” instances). Then, we shuffled 1000 times the DBP/C sites within the genomic space defined by the union of all genes that had participated in the correlations. In each of the 1000 iterations, we counted the instances of overlap with the specific gene set. After all iterations, we built a distribution of the expected number of overlaps with DBP/C sites. We compared the observed number of instances with this distribution and calculated the *Z*-score of the observed instances. We consider an absolute *Z*-score >10 as statistically significant. We include all the calculated *Z*-scores in Supplementary Data 5.

To evaluate the overlaps of MIR elements with DBP/Cs, we repeated the above simulations but instead of genes we focused our attention on the MIR elements embedded in the introns of the respective gene sets. Thus, for each of the 1000 iterations and for each of the “observed” instances, we counted the overlap of the DBP/Cs (or their shuffled binding peaks) with the respective MIR elements. *Z*-score calculations and significance thresholds were done in the same manner.

### Testing the genes’ architecture

We tested the architecture of gene sets as compared to the respective background, i.e., all genes participating in the respective analysis (for example, all genes that were expressed and considered for differential expression). For exonic and intronic length, we counted the number of bases in the union of exons or introns, respectively. For GC content, we counted G or C bases in the genes’ exons or introns and divided by the exons’ or introns’ length, respectively. We calculated the evolutionary conservation of a gene’s exons or introns as the median phyloP score of all exonic or intronic bases, respectively. For repetitive elements (e.g., Alu, MIR, L1, ERV elements), we estimated the fraction (density) of a gene’s exons or introns overlapping with each respective repetitive element. To evaluate statistically significant differences, we used Kolmogorov–Smirnov test. For repetitive elements, we also used Hypergeometric tests to evaluate whether the gene set under question contained genes that were more likely to contain at least one instance of each repetitive element as compared to the background gene population. For both tests, our default *p* value threshold was 10^−4^ but we also note cases were the *p* value was larger than 10^−4^ but lower than 10^−2^. The evolutionary conservation of the mCpGs and their probability of being part of a repetitive element or enhancer were tested with Kolmogorov-Smirnov or hypergeometric tests, respectively, against all mCpGs that participated in the correlations.

### Distances among expression profiles

We projected the expression profiles from the Glass et al. study^5^ on the samples from normal human hematopoiesis^29^. In detail, we first intersected the two data matrices and kept the genes expressed in both (*n* = 4839). Then, we rank-normalized the expression profiles with higher expression values ranked highest.

We performed principal component analysis on the rank-normalized matrix using only the normal samples. Then, we used the computed eigenvectors to project the leukemic samples on the reduced space. To aid the visualization, we fitted ellipses with the *car* package in R, at 0.8 confidence level for each lineage or AML subtype. We grouped: B, T, NK and NKT cells as “lymphoid”; basophils, dendritic, eosinophils, granulocytes and monocytes as “myeloid”; erythroid cells and megakaryocytes as “erythroid”; CMP, GMP and MEP cells as “progenitor”.

Using the rank-normalized matrix, we also computed the Manhattan distance between each leukemic expression profile (sample) and each normal one. This created a matrix of 211 rows (the normal profiles) and 36 columns (the leukemic profiles) with the values being the Manhattan distances of the respective profiles. We normalized this matrix so the median distance of each leukemic profile to all normal profiles is zero. We used this matrix to perform SAM and compute significantly different distances in the double mutants to each of the single mutant groups separately. On the normal profiles that SAM returned as significant, we used hypergeometric tests to look for enrichments in specific cell types with reference to all 211 cells and all 15 cell types and corrected the resulting *p* values to FDR values.

### Reporting summary

Further information on research design is available in the Nature Research Reporting Summary linked to this article.

## Supplementary information

Supplementary Information

Description of Additional Supplementary Files

Supplementary Data 1

Supplementary Data 2

Supplementary Data 3

Supplementary Data 4

Supplementary Data 5

Supplementary Data 6

Reporting Summary

## Data Availability

The publicly available datasets analyzed during the current study are available in the GEO repository under accession codes GSE86952^5^, GSE6891^47^, GSE24759^29^, GSE60055^6^, GSE104404^26^, GSE45144^21^, the GDC portal (https://gdc.cancer.gov/about-data/publications/laml_2012)^1^, the UCSC Genome Browser (http://genome.ucsc.edu/)^62^ for the ENCODE (http://genome.ucsc.edu/cgi-bin/hgTrackUi?hgsid=750328547_9j5VLyHWUZPZz7rzBYImG9AyNaZC&c=chr11&g=encRegTfbsClustered)^22^ and phyloP scores (http://hgdownload.soe.ucsc.edu/goldenPath/hg19/phyloP46way/vertebrate)^51^, RepeatMasker (https://www.repeatmasker.org/)^63^, KEGG (https://www.genome.jp/kegg/)^50^ and PICKLE (http://pickle.gr/). The Hi-C data generated for this study were deposited in GEO with accession code GSE188940. The remaining data are available within the Article, Supplementary Information or Source Data file. Source data are provided with this paper.

## Source data

Source Data

## Code availability

All custom code used in this study is available at https://github.com/atelonis/MIRs-IDH12-DNMT3A-AMLs_2022 and the corresponding DOI is as follows: 10.5281/zenodo.7072736.
