# Supplementary material for: MIR retrotransposons link the epigenome and the transcriptome of coding genes in acute myeloid leukemia
Source: Nat Commun. 2022 Oct 31;13:6524. doi: 10.1038/s41467-022-34211-x (PMC9622910; doi:10.1038/s41467-022-34211-x)
Supplement: Supplementary file 1 — Supplementary Information [file 41467_2022_34211_MOESM1_ESM.pdf]

Supplementary Information for

MIR Retrotransposons Link the Epigenome and the Transcriptome of Coding Genes in Acute Myeloid Leukemia

Aristeidis G. Telonis[1,2], Qin Yang[1,2] *, Hsuan-Ting Huang[1,2] *, Maria E. Figueroa[1,2]

[1]Department of Biochemistry and Molecular Biology, University of Miami Miller School of Medicine, Miami, Florida, USA
[2]Sylvester Comprehensive Cancer Center, University of Miami Miller School of Medicine, Miami, Florida, USA
* Contributed equally

Mailing Address:
1501 NW 10th Ave
Biomedical Research Building, Suite 709A
Miami, FL 33136
Tel.: 305-243-7333

Keywords: Acute Myeloid Leukemia; DNMT3A; TET2; IDH; Retrotransposon; Epigenetics

Supplementary Figure 1

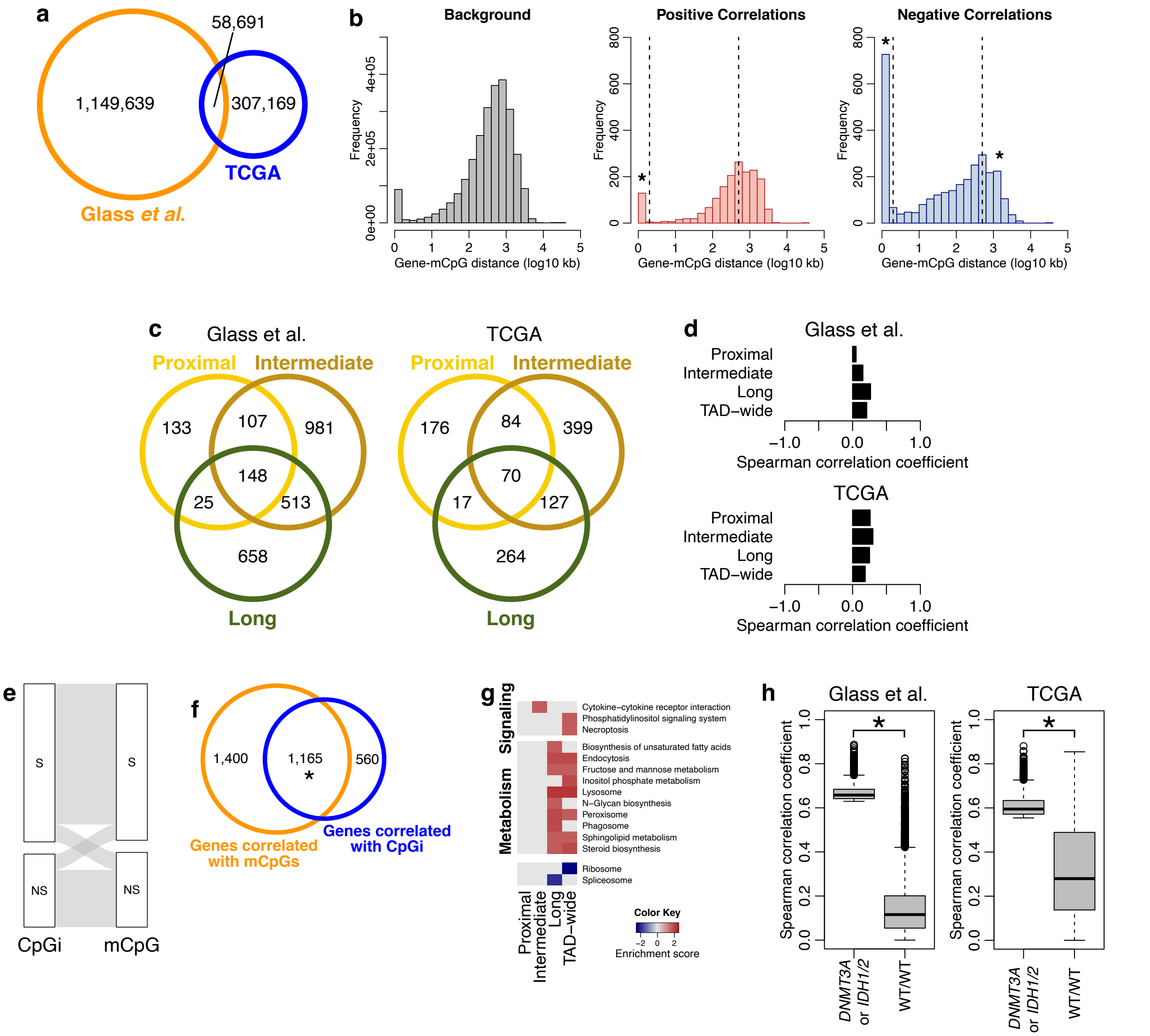

Supplementary Figure 1. Characteristics of the genes with significant expression-methylation correlations. (**a**) Venn diagram showing the number of CpGs captured by each cohort. DNA methylation was assessed in TCGA[1] by Illumina's methylation array while in Glass *et al*.[2] by enhanced reduced representation bisulfite sequencing (ERRBS). (**b**) Histograms of the distance between the mCpG and the gene for all pairs tested (left) and for the significant positive (middle) and negative (right) correlations in the TCGA cohort. The dashed vertical lines indicate the cutoffs for calling correlations as proximal, intermediate- or long-range. Asterisks indicate statistically significant enrichments in the positive (n=1,744; P-value$<10^{-10}$) or negative (n=2,992; P-value$<10^{-10}$) proximal correlations (Chi-squared test; one sided) or depletion in the long-range correlations ($P<10^{-2}$; Chi-squared test). (**c**) Venn diagram showing the overlap in the gene sets correlated with mCpG. (**d**) Spearman correlation coefficients of the number of mCpGs

significantly correlated with a gene with the number of all mCpGs tested for that gene (n=9,961 for Glass *et al*. and n=9,854 for TCGA). (**e**) Alluvial plot visualizing the percentage of CpGi (n=421; left column) that contain at least one mCpG (right column) with significant correlations to gene(s). S: the CpGi or mCpG has significant correlation(s); NS: the CpGi or mCpG has no significant correlation(s) to gene expression. (**f**) Venn diagram showing the number of genes significantly correlated with mCpGs or with CpG islands (CpGi). The asterisk indicates a statistically significant overlap (P-value<10^{-10}; one-sided Hypergeometric test). (**g**) Heatmap showing the pathways enriched or depleted from the correlation analysis of CpGi to gene expression as per GSEA. The genes were ranked by absolute maximum correlation coefficient with CpGi methylation. (**h**) Box plot showing the distribution of the correlation coefficients that are significant in the Glass *et al*. and TCGA cohort of samples with *IDH1/2* or *DNMT3A* mutations (left box) or the same coefficients when examined the TCGA samples with no mutations in these genes (WT/WT; right box). Asterisks indicate statistical significance, P-value<10^{-4}, two-sided Mann-Whitney U-test. Box plots show the median (center), 25–75 percentile (box), and 5–95 percentile (whisker) from n=10,651 and 4,071 correlations in Glass *et al.* and TCGA, respectively.

Supplementary Figure 2

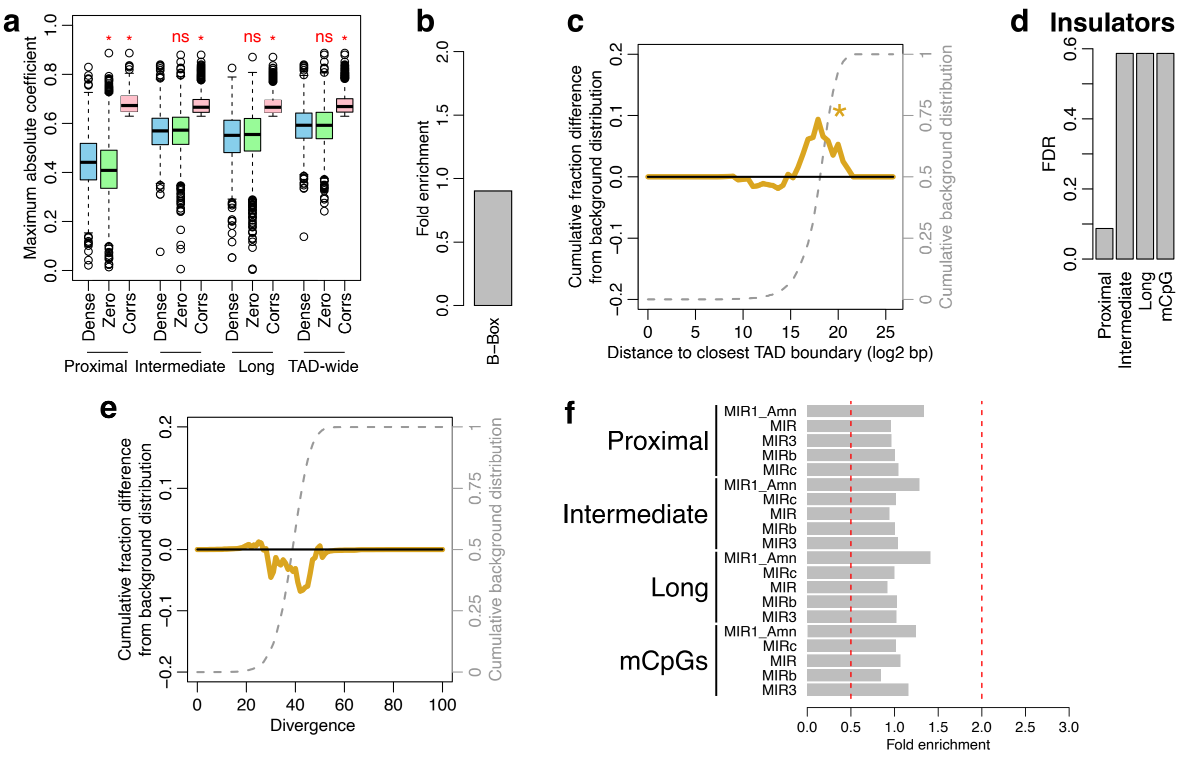

Supplementary Figure 2. Properties of the MIR elements. (**a**) Box plots showing the distribution of the maximum absolute correlation coefficient of each gene with the mCpG in the same TAD (also referred as the methylation dependency of each gene) per distance bin. The box marked as "Dense" refers to the top 10% genes (n=882 genes) as ranked by intronic MIR density. The box marked as "Zero" refers to the genes with no MIRs in their introns but with similar CpG density as the MIR-dense genes (CpG density within one standard deviation of the mean of the respective MIR-dense genes; on average 1885 genes). The box marked as "Corrs" refers to the genes with significant expression-methylation correlations in the respective distance bin. Asterisks mark statistically significant difference compared to the "Dense" box (P-value<0.01; two-sided Mann-Whitney U-test); ns: non-significant difference (P-value>0.01). Box plots show the median (center), 25–75 percentile (box), and 5–95 percentile (whisker). (**b**) Barplot showing the enrichment of MIR elements containing at least one B-box (GTTCNANNC) in sense or antisense orientation in the MIRs overlapping mCpG with significant correlations. (**c**) Plot showing the distance of the n=144 MIRs overlapping mCpG with significant correlations to the closest TAD boundary as compared to the respective background of MIRs overlapping all tested mCpG. Asterisk indicates P-value=0.05064 per Kolmogorov-Smirnov test (two-sided). (**d**) FDR for the enrichment of MIR insulators (n=56 for gene sets; n=6,008 for mCpGs) in the MIRs with overlapping genes or mCpGs with significant

correlations; P-values were calculated with hypergeometric tests (one-sided). (**e**) Plot showing the divergence score of the MIRs overlapping mCpG with significant correlations (n=144) as compared to the respective background MIRs overlapping all tested mCpG. P-value=0.371; Kolmogorov-Smirnov test (two-sided). (**f**) Barplot showing the fold enrichment scores of MIR subfamilies in the MIRs overlapping genes or mCpG with significant correlations as compared to the MIRs overlapping all background genes or tested mCpG.

Supplementary Figure 3

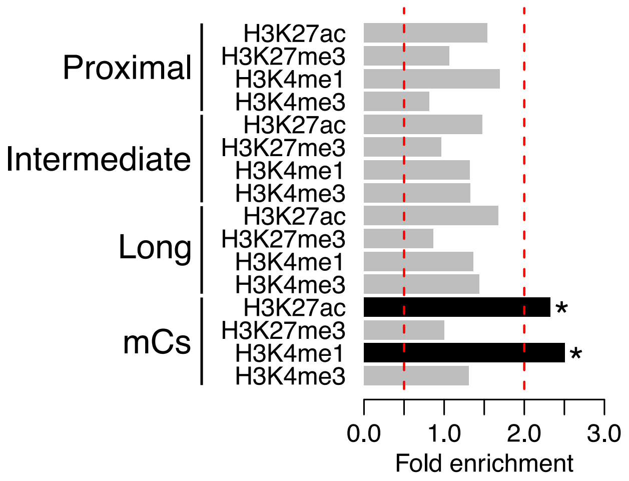

Supplementary Figure 3: Histone marks on MIR elements. Overlap of histone marks in CD34+ CD38- cells from Adelman *et al.* with the MIR elements of genes (n=56) or mCpG (n=144) with significant correlations. Asterisks indicate statistically significant overlap (FDR<5%; one-sided Hypergeometric test) as compared to the background set of MIRs (all MIRs overlapping the tested genes or mCpG, respectively).

SUPPLEMENTARY REFERENCES

1. Cancer Genome Atlas Research, N. *et al.* Genomic and epigenomic landscapes of adult de novo acute myeloid leukemia. *N Engl J Med* **368**, 2059-74 (2013).
2. Glass, J.L. *et al.* Epigenetic Identity in AML Depends on Disruption of Nonpromoter Regulatory Elements and Is Affected by Antagonistic Effects of Mutations in Epigenetic Modifiers. *Cancer Discov* **7**, 868-883 (2017).
