# Supplementary material for: MIR retrotransposons link the epigenome and the transcriptome of coding genes in acute myeloid leukemia
Source: Nat Commun. 2022 Oct 31;13:6524. doi: 10.1038/s41467-022-34211-x (PMC9622910; doi:10.1038/s41467-022-34211-x)
Supplement: Supplementary file 2 — Description of Additional Supplementary Files [file 41467_2022_34211_MOESM2_ESM.pdf]

Description of Additional Supplementary Files

File Name: Supplementary Data 1
Description: Topologically associated domains (TADs) in human CD34+ cells

File Name: Supplementary Data 2
Description: Correlations, differentially expressed genes & differentially methylated cytosines

File Name: Supplementary Data 3
Description: Pathway analyses in correlations or differentially expressed genes

File Name: Supplementary Data 4
Description: Gene architecture biases

File Name: Supplementary Data 5
Description: Enrichment/Depletion of histone marks and Transcription Factors.

File Name: Supplementary Data 6
Description: Integrative analysis of leukemic samples and normal cells.
