# Supplementary material for: MIR retrotransposons link the epigenome and the transcriptome of coding genes in acute myeloid leukemia
Source: Nat Commun. 2022 Oct 31;13:6524. doi: 10.1038/s41467-022-34211-x (PMC9622910; doi:10.1038/s41467-022-34211-x)
Supplement: Supplementary file 9 — Reporting Summary [file 41467_2022_34211_MOESM9_ESM.pdf]

nature portfolio

Corresponding author(s): Maria Eugenia Figueroa

Last updated by author(s): 17 October 2022

Reporting Summary

Nature Portfolio wishes to improve the reproducibility of the work that we publish. This form provides structure for consistency and transparency in reporting. For further information on Nature Portfolio policies, see our Editorial Policies and the Editorial Policy Checklist.

Statistics

For all statistical analyses, confirm that the following items are present in the figure legend, table legend, main text, or Methods section.

n/a	Confirmed	
☐	☒	The exact sample size (*n*) for each experimental group/condition, given as a discrete number and unit of measurement
☒	☐	A statement on whether measurements were taken from distinct samples or whether the same sample was measured repeatedly
☐	☒	The statistical test(s) used AND whether they are one- or two-sided<br>*Only common tests should be described solely by name; describe more complex techniques in the Methods section.*
☐	☒	A description of all covariates tested
☐	☒	A description of any assumptions or corrections, such as tests of normality and adjustment for multiple comparisons
☐	☒	A full description of the statistical parameters including central tendency (e.g. means) or other basic estimates (e.g. regression coefficient) AND variation (e.g. standard deviation) or associated estimates of uncertainty (e.g. confidence intervals)
☐	☒	For null hypothesis testing, the test statistic (e.g. *F*, *t*, *r*) with confidence intervals, effect sizes, degrees of freedom and *P* value noted<br>*Give P values as exact values whenever suitable.*
☒	☐	For Bayesian analysis, information on the choice of priors and Markov chain Monte Carlo settings
☐	☒	For hierarchical and complex designs, identification of the appropriate level for tests and full reporting of outcomes
☒	☐	Estimates of effect sizes (e.g. Cohen's *d*, Pearson's *r*), indicating how they were calculated

Our web collection on statistics for biologists contains articles on many of the points above.

Software and code

Policy information about availability of computer code

Data collection	All statistical analyses were conducted in python version 2.7.16 or R version 3.6.1. We made use of the numpy (v. 1.16.5) and scipy (v. 0.19.1) packages in python and amap (v.0.8-18), circlize58 (v. 0.4.12), data.table (v. 1.3.16), dendextend (v, 1.14.0), methylSig (v. 0.5.2) samr (v. 3.0) and VennDiagram (v. 1.6.20) packages in R as well as Cytoscape version 3.7.261. All custom code used in this study is available at https://github.com/atelonis/MIRs-IDH12-DNMT3A-AMLs_2022 and with DOI: 10.5281/zenodo.7072736.
Data analysis	All statistical analyses were conducted in python version 2.7.16 or R version 3.6.1. We made use of the numpy (v. 1.16.5) and scipy (v. 0.19.1) packages in python and amap (v.0.8-18), circlize58 (v. 0.4.12), data.table (v. 1.3.16), dendextend (v, 1.14.0), methylSig (v. 0.5.2) samr (v. 3.0) and VennDiagram (v. 1.6.20) packages in R as well as Cytoscape version 3.7.261. All custom code used in this study is available at https://github.com/atelonis/MIRs-IDH12-DNMT3A-AMLs_2022 and with DOI: 10.5281/zenodo.7072736.

For manuscripts utilizing custom algorithms or software that are central to the research but not yet described in published literature, software must be made available to editors and reviewers. We strongly encourage code deposition in a community repository (e.g. GitHub). See the Nature Portfolio guidelines for submitting code & software for further information.

Data

Policy information about availability of data

All manuscripts must include a data availability statement. This statement should provide the following information, where applicable:

- Accession codes, unique identifiers, or web links for publicly available datasets
- A description of any restrictions on data availability
- For clinical datasets or third party data, please ensure that the statement adheres to our policy

The publicly available datasets analyzed during the current study are available in the GEO repository under accession codes GSE86952 [https://

www.ncbi.nlm.nih.gov/geo/query/acc.cgi?acc=GSE86952], GSE6891 [https://www.ncbi.nlm.nih.gov/geo/query/acc.cgi?acc=GSE6891], GSE24759 [https://www.ncbi.nlm.nih.gov/geo/query/acc.cgi?acc=GSE24759], GSE60055 [https://www.ncbi.nlm.nih.gov/geo/query/acc.cgi?acc=GSE60055], GSE104404 [https://www.ncbi.nlm.nih.gov/geo/query/acc.cgi?acc=GSE104404], GSE45144 [https://www.ncbi.nlm.nih.gov/geo/query/acc.cgi?acc=GSE45144] the GDC portal (https://gdc.cancer.gov/about-data/publications/laml_2012), the UCSC Genome Browser (http://genome.ucsc.edu/) for the ENCODE (http://genome.ucsc.edu/cgi-bin/hgTrackUi?hgsid=750328547_9j5VLyHWUZPZz7rzBYImG9AyNaZC&c=chr11&g=encRegTfbsClustered) and phyloP scores (http://hgdownload.soe.ucsc.edu/goldenPath/hg19/phyloP46way/vertebrate), RepeatMasker (https://www.repeatmasker.org/), KEGG (https://www.genome.jp/kegg/) and PICKLE (http://pickle.gr/). The Hi-C data generated for this study were deposited in GEO with accession code GSE188940 (https://www.ncbi.nlm.nih.gov/geo/query/acc.cgi?acc=GSE188940). Source data are provided with this paper. The remaining data are available within the Article, Supplementary Information or Source Data file.

Field-specific reporting

Please select the one below that is the best fit for your research. If you are not sure, read the appropriate sections before making your selection.

☒ Life sciences ☐ Behavioural & social sciences ☐ Ecological, evolutionary & environmental sciences

For a reference copy of the document with all sections, see nature.com/documents/nr-reporting-summary-flat.pdf

Life sciences study design

All studies must disclose on these points even when the disclosure is negative.

Sample size	We did not use any statistical test to estimate sample size. Sample size was estimated based on our previous experience with similar types of analyses.
Data exclusions	No data were excluded.
Replication	The findings were reproduced in two independent cohort (one cohort from the Figueroa lab and the TCGA cohort).
Randomization	Randomization is not relevant to this study as samples were not allocated into separate experimental groups.
Blinding	Investigators were not blinded to group allocation. Blinding was not possible as the same investigator downloaded and analyzed publicly available data. However, all samples were processed and analyzed in the same manner by the same computational algorithms.

Reporting for specific materials, systems and methods

We require information from authors about some types of materials, experimental systems and methods used in many studies. Here, indicate whether each material, system or method listed is relevant to your study. If you are not sure if a list item applies to your research, read the appropriate section before selecting a response.

Materials & experimental systems

n/a	Involved in the study	
☒	☐	Antibodies
☐	☒	Eukaryotic cell lines
☒	☐	Palaeontology and archaeology
☒	☐	Animals and other organisms
☒	☐	Human research participants
☒	☐	Clinical data
☒	☐	Dual use research of concern

Methods

n/a	Involved in the study	
☒	☐	ChIP-seq
☒	☐	Flow cytometry
☒	☐	MRI-based neuroimaging

Eukaryotic cell lines

Policy information about cell lines

Cell line source(s)	We analyzed publicly available data from K562 cells from ENCODE. On the ENCODE data portal each experiment is linked to a specific biosample page with details about the sample source.
Authentication	We analyzed publicly available data from K562 cells from ENCODE. On the ENCODE data portal each experiment is linked to a specific biosample page with details about the sample authentication.
Mycoplasma contamination	No testing for mycoplasma contamination was performed.
Commonly misidentified lines (See ICLAC register)	No commonly misidentified lines were used.
